# Achieving simultaneous removal of carbon and nitrogen by an integrated process of anaerobic membrane bioreactor and flow-through biofilm reactor

**DOI:** 10.1016/j.engmic.2023.100136

**Published:** 2023-12-15

**Authors:** Xueshen Wu, Chao Wang, Depeng Wang, Ahmed Tawfik, Ronghua Xu, Zhong Yu, Fangang Meng

**Affiliations:** a School of Environmental Science and Engineering, Sun Yat-sen University, Guangzhou 510006, China; b Guangdong Provincial Key Laboratory of Environmental Pollution Control and Remediation Technology, Guangzhou 510006, China; c National Research Centre, Water Pollution Research Department, 12622, Dokki, Cairo, Egypt; d College of Life Sciences, Environmental Sciences Department, Kuwait University, P.O. 5969, Safat 13060, Kuwait

**Keywords:** Anaerobic membrane bioreactors, Anammox, Bacterial community, Livestock wastewater, Methanogens, Nitrogen removal

## Abstract

In this study, a combined system consisting of an anaerobic membrane bioreactor (AnMBR) and flow-through biofilm reactor/CANON (FTBR/CANON) was developed to simultaneously remove carbon and nitrogen from synthetic livestock wastewater. The average removal efficiencies of total nitrogen (TN) were 64.2 and 76.4% with influent ammonium (NH_4_^+^-N) concentrations of approximately 200 and 500 mg/L, respectively. The COD removal efficiencies were higher than 98.0% during the entire operation. Mass balance analysis showed that COD and TN were mainly removed by the AnMBR and FTBR/CANON, respectively. The anammox process was the main nitrogen removal pathway in the combined system, with a contribution of over 80%. High functional bacterial activity was observed in the combined system. Particularly, an increase in the NH_4_^+^-N concentration considerably improved the anammox activity of the biofilm in the FTBR/CANON. 16S rRNA high-throughput sequencing revealed that *Methanosaeta, Candidatus Methanofastidiosum*, and *Methanobacterium* were the dominant methanogens in the AnMBR granular sludge. In the CANON biofilm, *Nitrosomonas* and *Candidatus* Kuenenia were identified as aerobic and anaerobic ammonium-oxidizing bacteria, respectively. In summary, this study proposes a combined AnMBR and FTBR/CANON process targeting COD and nitrogen removal, and provides a potential alternative for treating high-strength wastewater.

## Graphical abstract

**Figure:**
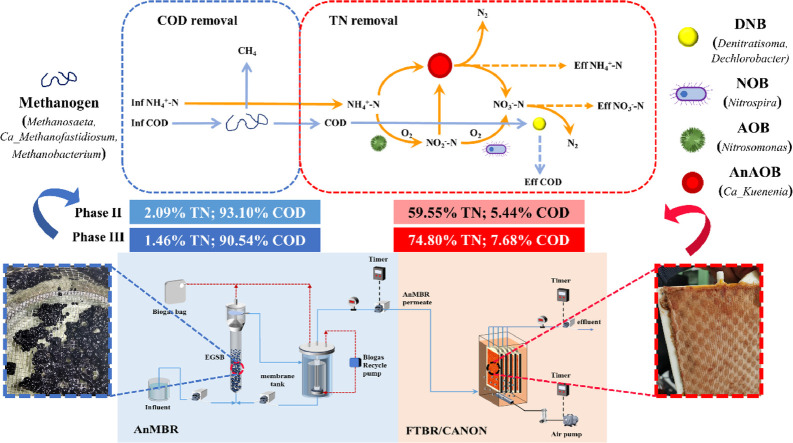

## 1 Introduction

It has been well-known that anaerobic membrane bioreactor (AnMBR) can achieve efficient organic matter removal and energy recovery [1]. Lab-, pilot-, and full-scale AnMBR have been widely used to treat domestic and high-strength wastewater [2], [3], [4]. However, owing to the lack of anoxic or aerobic zones, nitrogen removal in AnMBRs is negligible [5]. In most cases, AnMBRs are coupled with post-treatment processes to simultaneously remove organic matter and nutrients, such as microalgae photobioreactors [4]. For nitrogen-rich wastewater, such as livestock wastewater [6], landfill leachate [7], and dairy wastewater [8], autotrophic nitrogen removal is preferred for treating AnMBR effluent [9].

Capturing anaerobic ammonium oxidation bacteria (AnAOB) and aerobic ammonium oxidation bacteria (AerAOB) is a prerequisite for achieving autotrophic nitrogen removal. Owing to the extremely low growth rates of AnAOB and AerAOB, even a small amount of these bacteria loss may cause a sharp decline in nitrogen removal performance [10]. Therefore, several methods including granulation [11], biofilms [12], and membrane separation [13] have been developed to retain and enrich AnAOB and/or AerAOB in bioreactors. Among these, membrane bioreactors (MBRs) have shown great success in AnAOB and AerAOB enrichment and exhibit satisfactory nitrogen removal performance [14,15]. For instance, in a previous study, the use of MBRs resulted in a significant reduction in the doubling time of AnAOB [16]. However, high operating costs and the occurrence of membrane fouling strongly hinder the application of MBRs for autotrophic nitrogen removal [10].

Recently, several new MBR processes have been developed to overcome the disadvantages of conventional technology. One example is dynamic MBRs, which use low-cost filters, such as non-woven fibers or meshes, to replace membranes [17]. Dynamic membranes developed on filters can improve the physical rejection of bacteria and pollutants. Moreover, a flow-through biofilm reactor (FTBR) has been proposed, in which the biofilm plays two roles: dynamic membranes for solid/liquid separation and active biofilms for bioconversion [18]. We have found that AnAOB can be enriched in FTBR during long-term operation [19,20]. Nevertheless, the knowledge gap lies in the incorporation of AerAOB and AnAOB into a single FTBR biofilm, which has not been clearly explored. The combination of AnMBR and FTBR based on autotrophic nitrogen removal process, with a compact configuration and dynamic continuous operation, is expected to offer a promising alternative for the treatment of high-strength wastewater containing both organics and ammonium.

In this study, a laboratory-scale FTBR for complete autotrophic nitrogen removal during the nitrite process (FTBR/CANON) was developed. Additionally, to achieve the simultaneous removal of carbon and nitrogen, the FTBR/CANON was integrated with an AnMBR for the treatment of synthetic livestock wastewater. The performance and microbial activity of the AnMBR-FTBR/CANON process with different influent NH_4_^+^-N concentrations (200 mg/L and 500 mg/L) were systematically investigated. The microbial communities were analyzed using 16S rRNA gene sequencing. We believe that this novel combined process would provide a potential alternative for the energy-efficient treatment of high-strength wastewater (i.e., livestock wastewater).

## 2 Materials and methods

### 2.1 Setup and operation of the FTBR/CANON

**Fig. 1:**
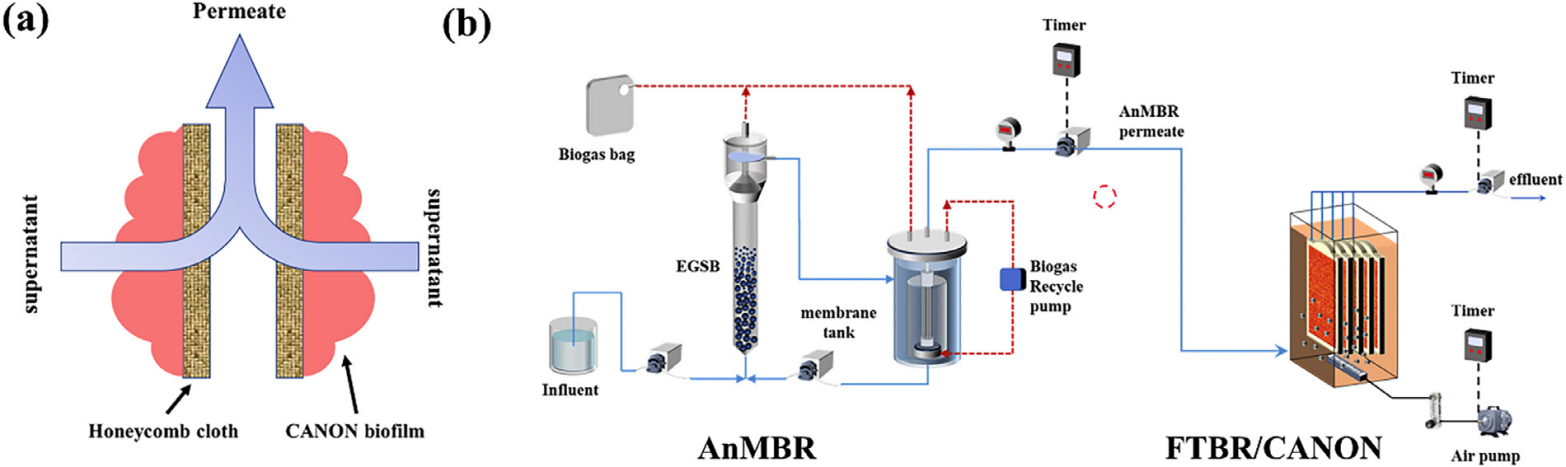
Flow-through mode of FTBR/CANON (a); schematic of the AnMBR-FTBR/CANON process (b).

Two treatment processes were employed to achieve the simultaneous removal of organics and nitrogen (Fig. 1). Before the operation of the combined process, the FTBR was operated alone (phase I) to incubate AerAOB and AnAOB, as well as to achieve the CANON process. The working volume of the FTBR unit was 5 L, and the hydraulic retention time (HRT) was set to 24 h. Six flat-sheet honeycomb cotton modules (0.1 m^2^, *L* × *W* × *H*: 32 × 22 × 1.4 cm^3^) were submerged in the reactor, and the total surface area was 0.6 m^2^. The thickness of each flat-sheet module was 0.4 cm, and the space between the two adjacent module surface was 0.5 cm. Anammox granular sludge (AMX-S, 500 mL) was collected from a laboratory-scale upflow column anammox reactor [21]. Furthermore, 500 mL of partial nitrification sludge (PN-S) was collected from a lab-scale PN-MBR that had been in operation for over 400 days [22]. The AMX-S and PN-S were mixed and crushed into small particles. Subsequently, six flat-sheet honeycomb cotton modules were submerged in the mixed liquor for 24 h to allow the mixed inoculant to deposit onto/into the fabrics.

After inoculating the AMX-S and PN-S, the FTBR was operated alone in phase I to obtain a stable CANON process (Table 1). The FTBR was continuously fed with wastewater containing NH_4_^+^-N (200 mg N/L). Trace elements are listed in Table S1. Unlike conventional biofilm reactors, the FTBR was operated in a flow-through mode (Fig. 1a). Thus, the treated wastewater must pass through the honeycomb cotton module and biofilms prior to discharge. The permeate of the FTBR was obtained from flat-sheet modules using a peristaltic pump. All the six modules were run at a considerably low flux of 0.35 L/(m^2^·h) (LMH) in an operation cycle of 8 min filtration/2 min relaxation [23]. The FTBR was operated at room temperature (20–25 °C). Intermittent aeration (5 min of aeration and 15 min of non-aeration) was used to achieve CANON in the FTBR. The aeration rate was controlled based on the NH_4_^+^-N concentration in the effluent. The changes of dissolved oxygen (DO) in one aeration/non-aeration cycle of the bulk FTBR/CANON reactor are shown in Fig. S1.

**Table 1:** Operation condition of AnMBR-FTBR/CANON.

Operating phases	Reactor	HRT (h)	Influent NH_4_^+^-N (mg-N/L)	Influent COD (mg/L)
Phase I (1–66 d)	FTBR/CANON	24	222.4 ± 26.1	0
Phase II (67–95 d)	AnMBR+FTBR/CANON	24+24	204.2 ± 60.6	1970.4 ± 180.0
Phase III (96–155 d)	AnMBR+FTBR/CANON	24+24	547.8 ± 51.3	1975.0 ± 132.0

### 2.2 Operation of AnMBR-FTBR/CANON (phases II and III)

A laboratory-scale external AnMBR was combined with the FTBR/CANON reactor in phases II and III (Table 1). The AnMBR in this study consisted of an expanded granular sludge bed (EGSB) column and an external ultrafiltration membrane tank (Fig. 1b). The effective volume of the EGSB column was 5 L. The EGSB reactor was inoculated with 1.5 L of anaerobic granular sludge obtained from a distillery, and the sludge bed height was consistently maintained at approximately 35 cm. The HRT of the EGSB reactor was set to 24 h. A hollow fiber membrane module (0.05 *μ*m pore size, polyvinylidene fluoride, Water code, Guangzhou, China) was submerged in the membrane tank. The operating flux of the AnMBR was set to 10 LMH (for more details of the AnMBR, see Table S2). Synthetic livestock wastewater was fed to the AnMBR to convert organic matter into CH_4_. The AnMBR permeate was directly fed into the FTBR/CANON to remove TN via partial nitrification-anammox (PN/A). The influent COD concentrations in phases II and III were set to 2000 mg/L, while the influent NH_4_^+^-N concentrations were 200 and 500 mg/L, respectively. No sludge was discharged from the two reactors during the 88 days of operation (phases II and III).

### 2.3 Measurements of specific rates of methanogenesis and anammox

The specific methanogenic activity (SMA) of the anaerobic granular sludge in the AnMBR (named as AnMBR-AnGS) was determined using an AMPTS II-methane potential analyzer (Bioprocess Control, Sweden) on days 88, 92, and 93 (phase II) and days 144, 148, and 151 (phase III). Further details on the SMA test were provided by Sun [24]. An in situ batch test was conducted to determine the specific anaerobic ammonium oxidation rate (SAA). The measurement method for the SAA is as follows: 1) The combined system was stopped and supernatant of the FTBR/CANON reactor was discharged. 2) An inorganic nitrogen medium (5 L) containing NH_4_^+^-N (50 mg N/L), NO_2_^−^-N (66 mg N/L), and PO_4_^3−^-P (10 mg P/L) was added to the reactor (NH_4_^+^-N:NO_2_^−^-*N* = 1:1.32). The pH of the inorganic nitrogen medium was also controlled at approximately 8.0 by adding Na_2_CO_3_. During the batch tests, a peristaltic pump was used to recirculate the reactor bulk. Water samples were collected every hour, and then filtered through a 0.45-*μ*m filter (Millipore, PES). The concentrations of NH_4_^+^-N, NO_2_^−^-N, and NO_3_^−^-N were analyzed. The SAA of the biofilms in the FTBR/CANON (CANON-biofilm) reactor was calculated using Eq (1). SAA tests were conducted on days 57, 58, and 59 (phase I), days 89, 90, and 91 (phase II), and days 140, 141, and 142 (phase III).

(1) $$SAA\;(\text{mg}-N/(m^{2}•h)=\frac{k•V}{A}$$

Where *k* refers to the maximum linear regression slope (mg-N/(L·h)) of NH_4_^+^-N concentration-time curve, *V* is the effective volume of the culture solution (5 L), and *A* represents the total surface area of the modules in the FTBR/CANON (0.6 m^2^).

### 2.4 Physicochemical analysis

The influent, AnMBR permeate, CANON supernatant, and CANON permeate were regularly collected. The water samples were filtered through a 0.45-*μ*m filter (Millipore, PES) after collecting from the reactors. COD, NH_4_^+^-N, nitrite nitrogen (NO_2_^−^-N), and nitrate nitrogen (NO_3_^−^-N) were measured using standard methods [25]. The removal efficiencies of NH_4_^+^-N, TN, and COD were calculated according to a method proposed by Wang [26]. The DO concentrations of the FTBR/CANON were monitored using a digital portable meter (WTW, Multi 3510 IDS, Germany). The turbidity of the FTBR/CANON (supernatant and permeate) was determined using a turbidimeter (WGZ-1B, Qiwei, Hangzhou, China). The pH, water temperature, and biogas composition were determined as previous report [27]. Free ammonia (FA) and free nitrous acid (FNA) were calculated using Eqs. (2) and (3), respectively, which were defined by Zhang [28].

(2) $$FA(\text{mg}/L)=\frac{{\left[NH_{4}^{+}-N\right]}_{\text{sup}}\times 10^{pH}}{\text{exp}[-\frac{6334}{273+T}]+10^{pH}}$$

(3) $$FNA(\mu g/L)=\frac{1000\;\times {\left[NO_{2}^{-}-N\right]}_{\text{sup}}}{\text{exp}[-\frac{2300}{273+T}]\;\times 10^{pH}}$$

Where [NH_4_^+^-N]_sup_ and [NO_2_^−^-N]_sup_ denote the concentrations of NH_4_^+^-N and NO_2_^−^-N in the CANON supernatant, respectively, and pH and T denote the pH and water temperature ( °C) in the reactor bulk, respectively.

The morphologies of the AnMBR-AnGS and CANON-biofilms were observed by scanning electron microscopy (SEM, Quanta 400FEG, FEI, USA). An energy-dispersive X-ray analyzer (EDX, INCA, UK) was also employed to characterize the inorganic components of the AnMBR-AnGS and CANON-biofilms. A fluorescence spectrophotometer (F-4700, Hitachi, Japan) was used to characterize the dissolved organic matter (DOM) in the combined system. During the measurement, the emission wavelengths (Em) were detected from 200 to 550 nm in steps of 5 nm, while the excitation wavelengths (Ex) were set from 200 to 400 nm in steps of 5 nm. The scan speed was set to 30,000 nm/min. Moreover, parallel factor (PARAFAC) modeling was fitted using MATLAB R2019a with the DOMFluor toolbox, which could statistically analyze the three-dimensional excitation-emission matrix (3D-EEM) datasets. GraphPad Prism 9.3.0 and Origin 2021 were used to visualize the PARAFAC results.

### 2.5 Mass balance assessment of carbon and nitrogen transformation

To assess the relative contributions of the two reactors to carbon and nitrogen removal, the mass balances of carbon and nitrogen were analyzed based on long-term observations of the AnMBR-FTBR/CANON system. The mass balance was calculated based on the following assumptions: 1) All of the COD removed in the FTBR/CANON was used for denitrification (NO_3_^−^→N_2_); 2) The contribution of assimilation to nitrogen loss was negligible, thus ignored. The details of the mass-balance calculations can be found elsewhere [28,29]. Based on Eq. (3), the S-K model proposed by Pandey [30] was used to evaluate the maximum TN removal rate (*U*_max_) of the FTBR/CANON.

(4) $$\begin{array}{c} \frac{\mathrm{HRT}}{TN_{\text{inf}}-TN_{\text{per}}}=\frac{K_{b}}{U_{\text{max}}}\frac{\mathrm{HRT}}{TN_{\text{inf}}}+\frac{1}{U_{\text{max}}} \end{array}$$

Where *TN*_inf_ and *TN*_per_ represent the TN concentrations in the influent and permeate of the FTBR/CANON, respectively, *HRT* represents the HRT of the FTBR/CANON (24 h), *K*_b_ is the saturation constant (mg-N/(L·h)), and *U*_max_ represents the maximum TN removal rate.

### 2.6 16S rRNA gene amplicon sequencing, processing, and statistical analysis

Twenty seeding sludge and biomass samples were collected from the AnMBR and FTBR/CANON for DNA extraction. A FastDNA Soil Kit (MP Biomedicals, CA, USA) was used to extract DNA from all samples according to the manufacturer's instructions. The primers 515F (5′-GTGCCAGCMGCCGCGGTAA-3′) and 806R (5′-GGACTACHVGGGTWTCTAAT-3′) were used to amplify the V4 region of the 16S rRNA gene [12]. PCR amplification and Illumina NovaSeq sequencing were performed by Guangdong Magigene Biotechnology Co. Ltd. (Guangdong, China). All raw sequencing data in this study have been deposited in the NCBI Sequence Read Archive database with the accession number PRJNA977399. Raw sequences were processed within the QIIME2 pipeline (2020.06) using the DADA2 method to generate amplicon sequence variants (ASVs). The sequences were rarefied to the lowest sequencing depth of 61,783 sequences per biomass sample. Representative ASV sequences were classified using the fitted classifier in the SILVA SSU database (version 138) with a confidence threshold of 80%.

Both the calculation of *α*-diversity index and *β*-diversity analysis were performed using the rarefied table using *R* software (v 4.2.2). The Bray–Curtis distances among different biomass samples were calculated using the “Vegan” package to represent the taxonomic *β*-diversity (principal coordinate analysis, PCoA). In addition, analysis of similarities (ANOSIM) was performed to determine the differences between the bacterial communities.

## 3 Results and discussion

### 3.1 Performance of the AnMBR-FTBR/CANON process

**Fig. 2:**
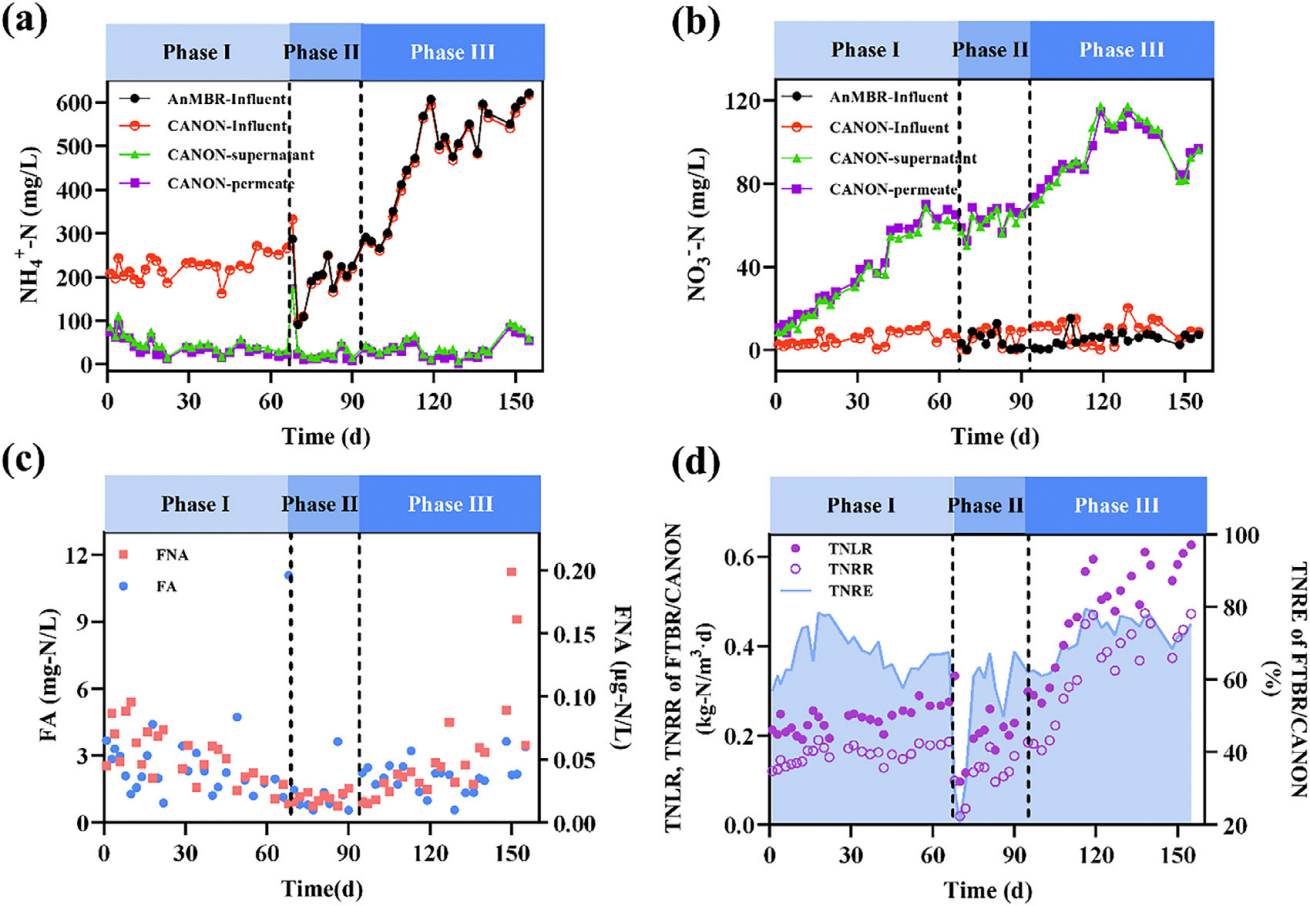
Concentrations of NH_4_^+^-N (a); NO_3_^−^-N (b); FA and FNA (c); and TNRR, TNLR and TNRE (d) of the AnMBR-FTBR/CANON process.

In phase I, the FTBR/CANON was operated alone to enrich AerAOB and AnAOB in advance (Table 1). Due to the low activity of AerAOB, high NH_4_^+^-N concentrations were observed in the FTBR/CANON permeate (Fig. 2a). After 10 d of operation, the effluent NH_4_^+^-N concentration decreased below 30 mg/L and then remained stable. Because of the proliferation of nitrite oxidizing bacteria (NOB), the effluent NO_3_^−^-N concentration exhibited an increasing trend (Fig. 2b). The total average nitrogen removal efficiency (TNRE) and ammonia removal efficiency (ARE) in phase I were 64.2% and 87.8%, respectively, implying the successful startup of the CANON process in the FTBR.

From day 75 (phases II and III), the FTBR/CANON was combined with an AnMBR. In phase II, the effluent NH_4_^+^-N concentration in the combined system dropped below 20 mg/L during the stable operation period (75–95 days). Notably, the effluent NO_3_^−^-N concentrations were maintained at approximately 60–70 mg/L during phase II (Fig. 2b). Additionally, the ∆NO_3_^−^-N/∆NH_4_^+^-N ratio of the final effluent maintained at approximately 0.28 during in this period (Fig. S2), further indicating that the intermittent aeration and the selective inhibition of free ammonia (FA, Fig. 2a) prevented the excessive reproduction of NOB.

**Fig. 3:**
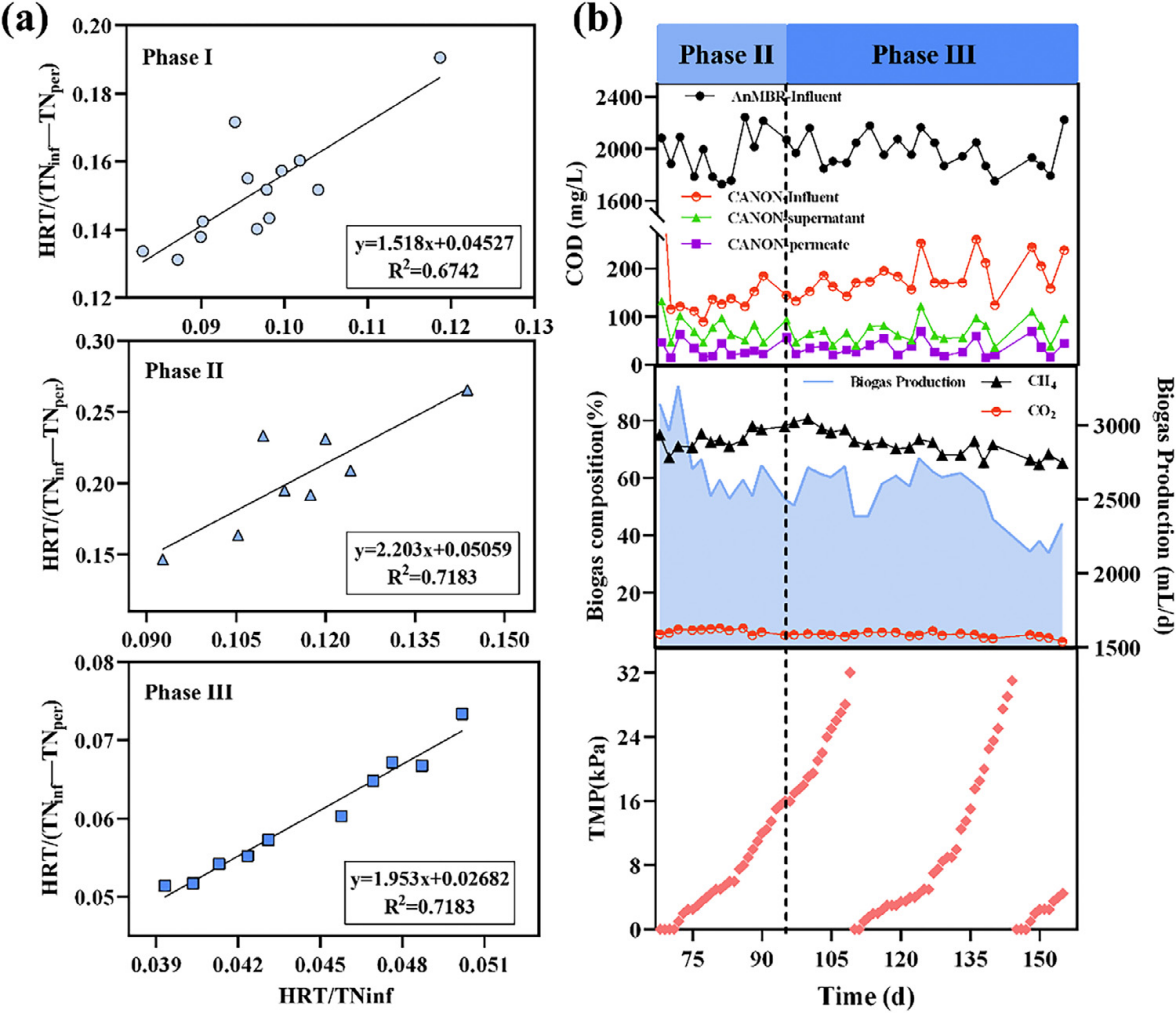
S-K model fitting of TN at different phases (column a); concentrations of COD, biogas composition, biogas production, and TMP of AnMBR-FTBR/CANON process (column b).

In phase III, the influent NH_4_^+^-N concentration gradually increased from approximately 200 to 500 mg-N/L. The average concentrations of effluent NH_4_^+^-N and NO_3_^−^-N were 15.8 and 106.9 mg-N/L during the stable operation in phase III, respectively. The ARE and TNRE were 96.8 and 76.4%, respectively. In phase III, the ∆NO_3_^−^-N/∆NH_4_^+^-N ratio of effluent was lower than that in phase II (average at 0.20, Fig. S2); however, it was still significantly higher than the theoretical value (0.11) [31]. No obvious accumulation of nitrite was observed in the FTBR/CANON during the entire operation, with NO_2_^−^-N and FNA concentrations below 4.0 mg-N/L (Fig. S2) and 0.1 *μ*g/L (Fig. 2c), respectively. According to the S-K model, the maximum TN removal capacity, *U*_max_, of the FTBR/CANON in phases I, II, and III was 22.1, 19.8, and 37.3 mg-N/(L·h), respectively (Fig. 3a). *U*_max_ in phase II was slightly lower than that in phase I, likely because of NOB proliferation.

The AnMBR exhibited a stable COD removal performance during the entire operation, except for slight fluctuations at the end of phase III (days 136–155) as the temperature decreased. In phases II and III, the average COD removal efficiency (Fig. 3b) of AnMBR was 93.3 ± 1.2% and 91.4 ± 1.3%, respectively (*P*>0.05). Similarly, no significant difference was found in the gas production (Fig. 3b) between the two phases (2612.5 ± 105.2 and 2614.3 ± 136.8 mL/d, respectively, *P*>0.05). Additionally, the biogas ratio of CH_4_ in the two phases was 74% (Fig. 3b). During the operation of the combined system, the membranes were chemically cleaned every 30–40 days (Fig. 3b); the membrane fouling rates were considerably lower than that of a submerged AnMBR in our previous studies [32], [33], benefiting from the external configuration of membrane units in this study. The heterotrophic bacteria and denitrifiers in the FTBR/CANON could further degrade the COD residue from the AnMBR. Thus, the effluent COD of combined system was as low as 32.6 ± 14.2 mg/L. The COD removal efficiency of the combined AnMBR-FTBR/CANON process in phases II and III were determined to be 98.5 ± 0.7% and 98.3 ± 0.6%, respectively (*P*>0.05).

The turbidity of the CANON supernatant varied significantly, ranging from 9.5 to 74.5 NTU, whereas the permeate turbidity of FTBR/CANON reactor remained below 1.5 NTU (Fig. S2). Moreover, over half of the supernatant COD in the FTBR/CANON reactor was rejected by non-woven fabrics and biofilms (average of 52.8%), resulting in a significantly lower permeate COD. These results imply that the CANON-biofilms could not only contribute to nitrogen conversion but could also reject and degrade considerable organics in the reactor bulk.

### 3.2 Carbon and nitrogen transformation pathways in the AnMBR-FTBR/CANON process

**Fig. 4:**
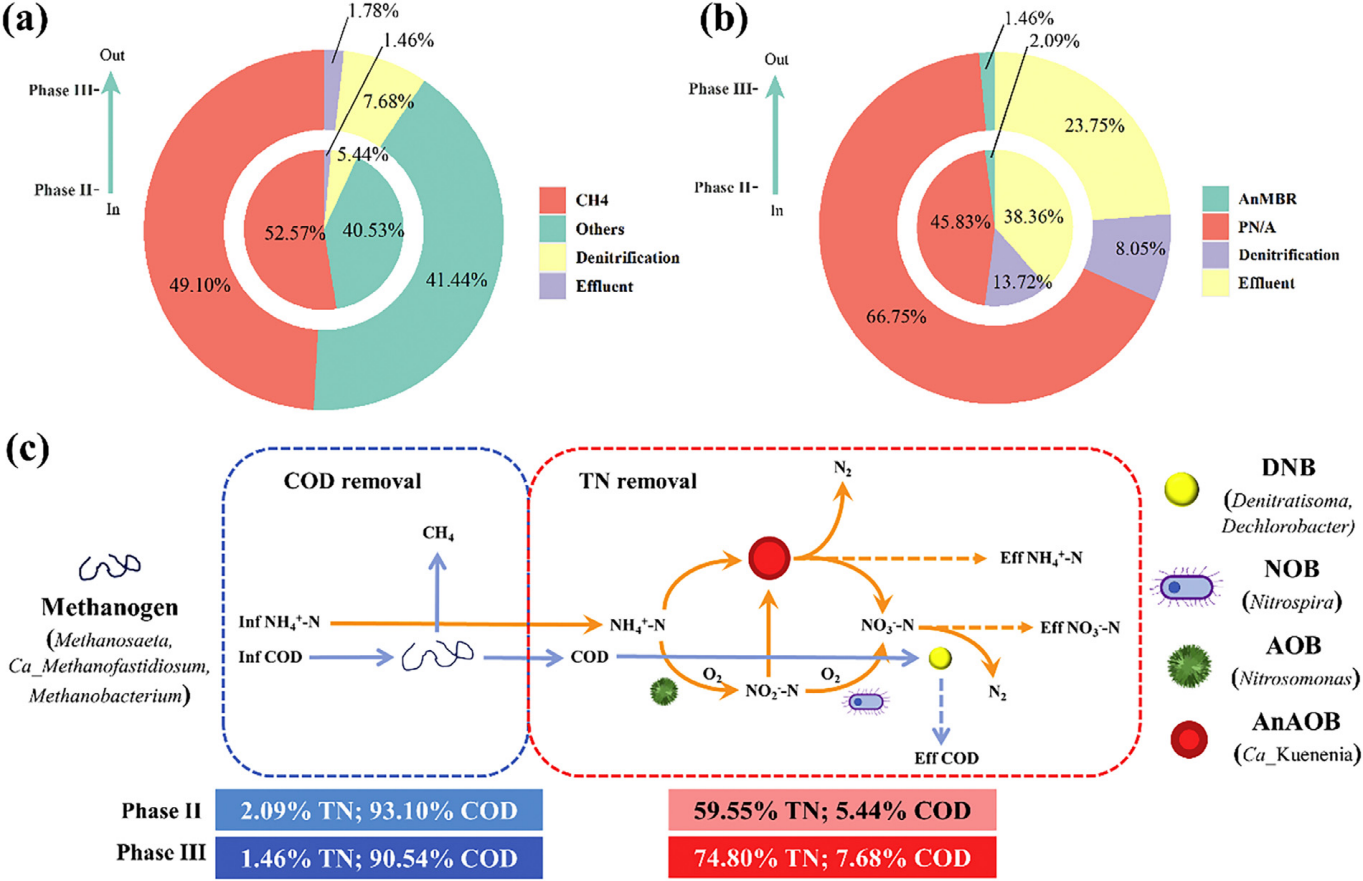
COD (a) and TN (b) balance of the AnMBR-FTBR/CANON system in phases II and III; and the mass flow of COD and TN (c).

Mass balance analysis of the AnMBR-FTBR/CANON was conducted to quantitatively characterize the carbon and nitrogen conversion pathways (Fig. 4). The COD conversion pathway exhibited slight variation between phases II and III. Approximately 50% of the influent COD was converted into gaseous CH_4_, whereas only 5.44–7.68% of the influent COD was utilized for denitrification in the FTBR/CANON (Fig. 4a). However, the conversion pathway for approximately 40% of the COD removal remains uncertain, likely due to bacterial growth and CO_2_ production. Rong et al. [29] investigated a pilot-scale AnMBR for municipal sewage treatment and observed that 18.1%, 26.7%, and 45.0% of the influent COD was transformed into dissolved CH_4_, excess sludge, and gaseous CH_4_, respectively. On average, 66.8% of the influent TN was removed by PN/A pathway in phase III, which was significantly higher than that removed in phase II (45.8%) (Fig. 4b). Autotrophic bacteria (AerAOB and AnAOB) appeared to play vital roles under high-ammonia conditions [34]. In general, anammox was the primary pathway for nitrogen removal in the combined AnMBR-FTBR/CANON system, accounting for over 80% of the TN removal. In the combined process, the contribution of AnMBR to COD removal accounted for 90.5–93.1% of the influent COD, whereas approximately 59.6–74.8% of the influent TN was removed by FTBR/CANON (Fig. 4c).

### 3.3 Variations of fluorescent DOM in the AnMBR-FTBR/CANON

**Fig. 5:**
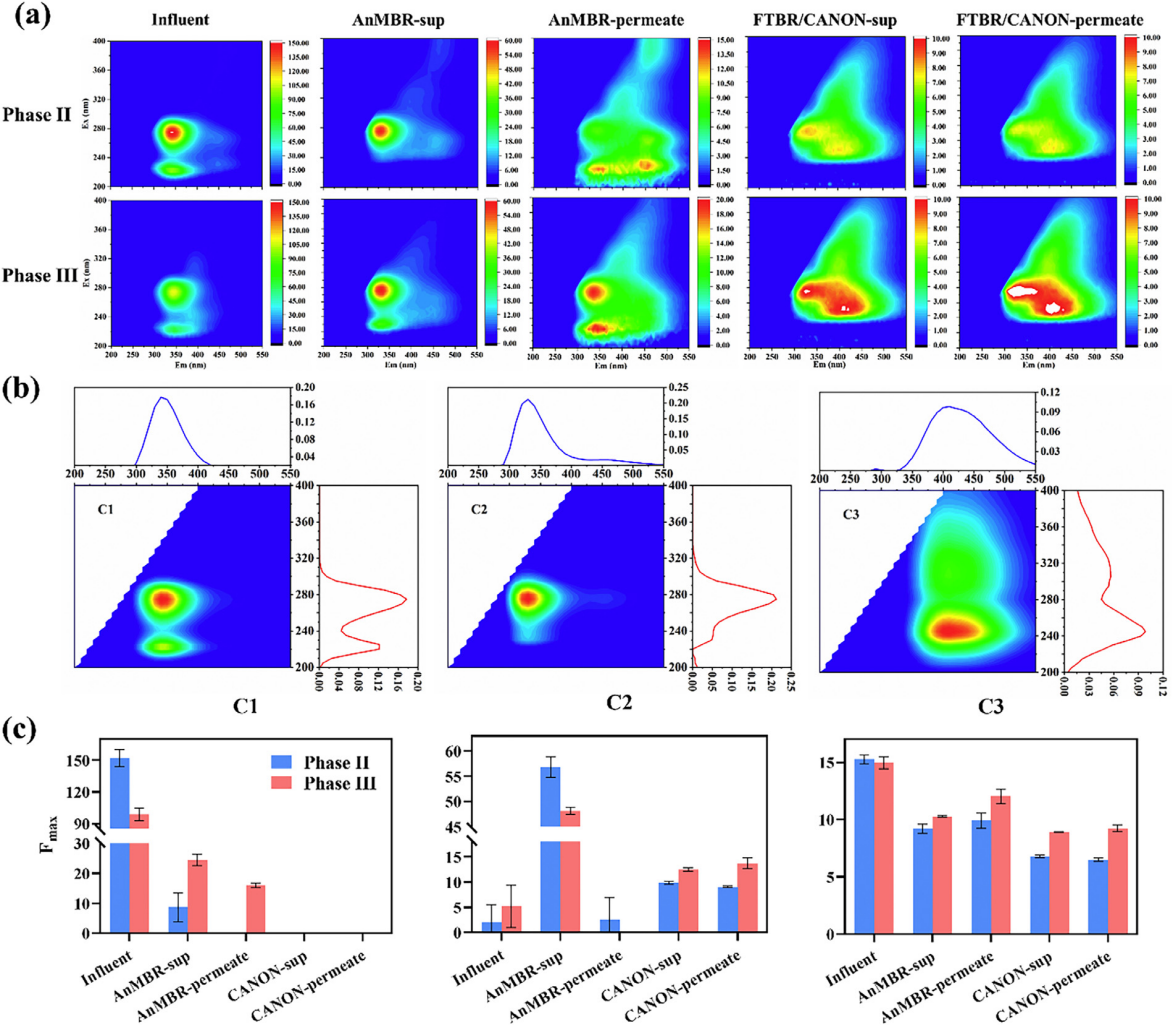
3D-EEM fluorescence spectra of DOM in the AnMBR-FTBR/CANON (a); PARAFAC modeling of the EEM spectra (b); variations of the three PARAFAC components in the AnMBR-FTBR/CANON (c).

3D-EEM spectroscopy was used to characterize the fluorescent DOM in the influent wastewater, AnMBR (supernatant and permeate), and FTBR/CANON (supernatant and permeate) (Fig. 5a). Three fluorescent DOM components, tryptophan-like (C1 and C2) and fulvic-like (C3) substances [35], were identified using PARAFAC modeling (Fig. 5b). The *F*_max_ of the C1 component in the influent wastewater was considerably high, likely due to the dosage of yeast extract in the influent (Table S1). The *F*_max_ of C1 decreased to nearly zero in the permeate of both reactors (Fig. 5c), implying that C1 was a readily biodegradable component [36]. In contrast to C1, *F*_max_ of C2 increased significantly in the permeate and supernatant of both reactors. Particularly, the *F*_max_ of C2 in the AnMBR supernatant (as well as the permeate) was significantly higher than that in the FTBR/CANON (*P*<0.01). Because the AnMBR was the main contributor to COD degradation, it was preliminarily determined that C2 was a type of protein secreted by heterotrophic bacteria [37]. The content of C2 was associated with the metabolic activity of heterotrophic bacteria in the AnMBR-FTBR/CANON system. Unlike the dynamic trends in C1 and C2, variations in C3 were insignificant in the combined treatment system. In phase III, *F*_max_ of C3 in FTBR/CANON was higher than that in phase II, attributable to the higher anammox activity in phase III (Fig. 5c). This was in line with the findings of Lu et al. [38], who reported that some fulvic-like substances (C3) were the main metabolites of AnAOB.

### 3.4 Metabolic activity and morphology of AnMBR-AGS and CANON-biofilm

**Fig. 6:**
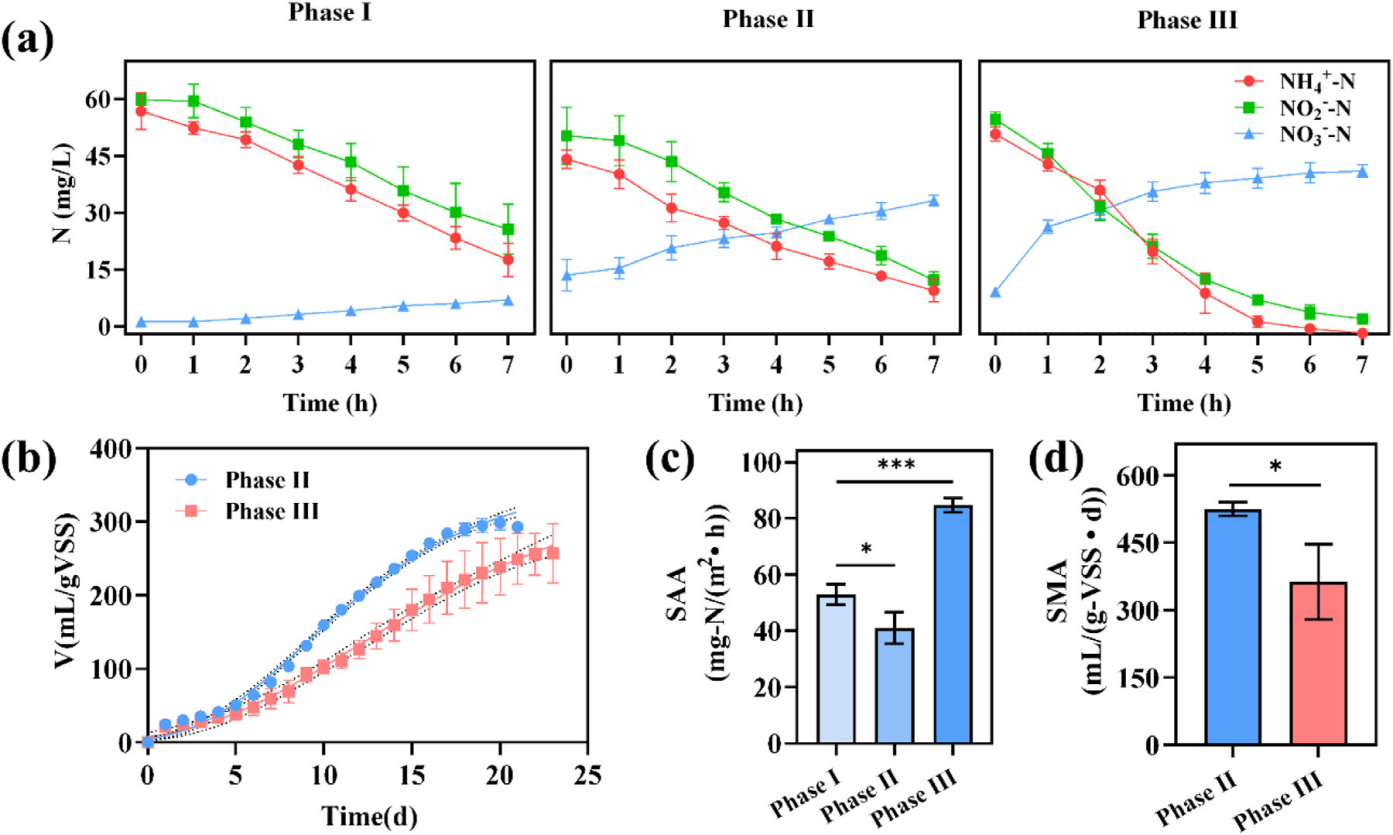
Variations of nitrogen concentrations (a) and CH_4_ production (b) during the batch tests; SAA (c) and SMA (d) of the AnMBR-FTBR/CANON process in different phases.

To explore the metabolic activity of functional bacteria (methanogens and AnAOB) in the AnMBR-FTBR/CANON, a series of batch tests were conducted. In phases I and II, the SAA of CANON-biofilm were determined to be 52.9 ± 3.66 and 41.0 ± 5.7 mg-N/(m^2^∙h), respectively (*P*<0.05) (Fig. 6c). The lower SAA levels in phase II were likely due to NOB proliferation. The SAA in phase III was significantly higher than that in phases I and II (84.6 ± 2.50 mg-N/(m^2^∙h), *P*<0.01), indicating the higher abundance or activity of AnAOB in phase III. Moreover, the SMA of the AnMBR-AGS in phases II and III were 525.1 ± 15.5 and 363.5 mL/(g-VSS∙d), respectively (*P*<0.05) (Fig. 6d). The decline in SMA in phase III might be related to the accumulation of inert matter in the reactor under operating conditions without biomass discharge. Yu et al. [39] stated that an increase in ash content in granular sludge weakens its affinity to substrates, thus leading to a decline in SMA. Additionally, higher FA in phase III than that in phase II could lead to a decrease in bacterial activity.

**Fig. 7:**
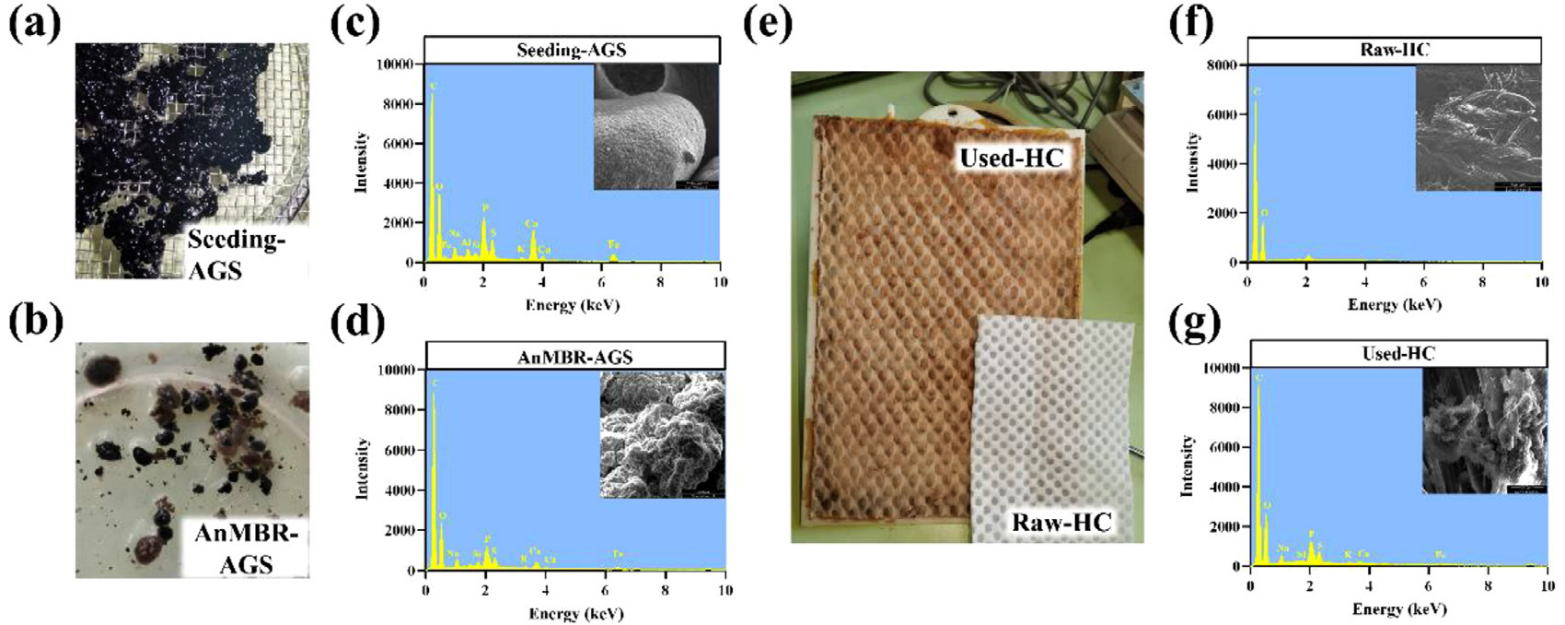
Photographs (a, b) and SEM-EDX images (c, d) of anaerobic granule sludge; photograph (e) and SEM-EDX images (f, g) of honeycomb cotton.

SEM and EDX images of the biomass are shown in Fig. 7. The surface of the seeding AGS was smooth and dense with no obvious cracks or EPS (Fig. 7c). Conversely, the surface of the AnMBR-AGS was rough and covered with a large amount of EPS secreted by anaerobic microorganisms (Fig. 7d). Furthermore, the EDX images showed that the proportion of inorganic elements in the AnMBR-AGS decreased significantly (5.5% vs. 11.0%) compared with the seeding AGS, owing to the low content of inorganic matter in the synthetic wastewater. The surface of the honeycomb cotton module was covered with considerable microbial aggregates (Fig. 7e). The structure of the biofilm on the cotton modules was relatively dense and mainly composed of cocci, filamentous bacteria, and EPS. The biofilm and honeycomb cotton were tightly wrapped together, indicating that honeycomb cotton had a strong ability to capture microorganisms. According to the EDX results, the raw honeycomb cotton modules contained only C and O, whereas the honeycomb cotton modules contained many inorganic elements related to microbial cells, such as S, P, Ca, K, and Fe. These results further corroborate that a large number of active microorganisms were enriched in honeycomb cotton during long-term operation.

### 3.5 Microbial diversity and functional microorganisms in the AnMBR-FTBR/CANON process

16S rRNA gene sequencing was used to determine the microbial diversity and functional microorganism composition in the AnMBR-FTBR/CANON system; 1389 ASVs were obtained from the 20 samples. The results of α-diversity analysis showed that the microbial diversity of AnMBR-AGS decreased slightly compared with that of seeding AGS (Table 2). The diversity of CANON-biofilm was higher than that of the seeding sludge of anammox (AMX-ss), but lower than that of the seeding sludge of partial nitritation (PN-ss) (Table 2). Additionally, the diversity of the CANON supernatant (CANON-sup) was slightly higher than that of the CANON-biofilm, probably due to the hydraulic conditions of the FTBR/CANON [40] or the enrichment of specific functional bacteria (AerAOB, AnAOB, NOB, etc.) in the CANON biofilm.

**Table 2:** α-diversity indices of different samples.

Samples	Number of ASVs	ACE	Chao1	Shannon	Simpson
Seeding-AGS	528	516.902	516.481	4.312	0.955
AnMBR-AGS	440	446.085	444.444	4.435	0.974
AMX-ss	285	315.866	316.954	3.267	0.895
PN-ss	638	581.397	583.202	3.740	0.922
CANON-sup	385	371.155	370.586	3.278	0.866
CANON-biofilm	282	329.854	329.579	3.348	0.889

* AMX-ss and PN-ss denote the seeding sludge from anammox and partial nitritation, respectively; CANON-sup and CANON-biofilm denote the bacterial sample from FTBR/CANON supernatant and biofilms, respectively.

**Fig. 8:**
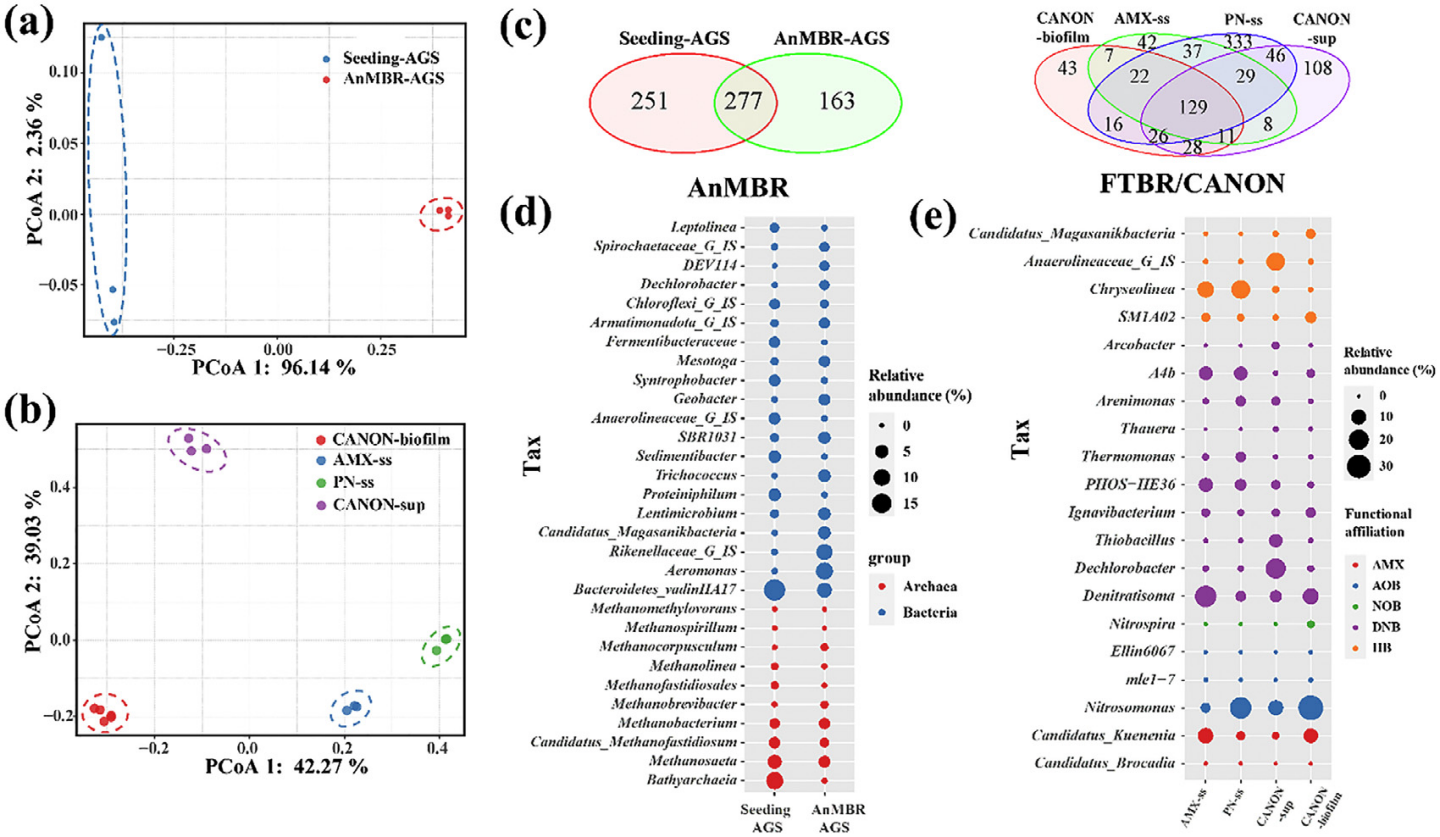
Venn diagram of ASVs obtained from the 20 communities (a); comparison of the microbial community structure (ASV) of the AnMBR-AGS (b) and CANON-biofilm (c) by PCoA according to the Bray–Curtis distance; Bubble plots representing the average relative abundance of key functional bacteria (genera level) in AnMBR-AGS (d) and CANON-biofilm (e).

A total of 509 genera were obtained from the 20 sequencing samples. Based on previous studies [4,41], the key functional bacteria are shown in Fig. 8. The PCoA results showed that the bacterial groups from the AnMBR and FTBR/CANON were clearly separated from each other (ANOSIM: *R* > 0.99, *P* = 0.001), suggesting that the microbial communities in the two reactors shifted significantly compared with those of the seeding sludge. As shown in the Venn diagram (Fig. 8c), the AnMBR-AGS and seeding AGS shared 277 ASVs, whereas the four samples in FTBR/CANON shared only 129 ASVs. In the seeding AGS community, the dominant archaea were *Bathyarchaeia* (10.87%), *Methanosaeta* (6.07%), *Candidatus methanofastidiosum* (3.03%), and *Methanobacterium* (2.17%). During long-term operation, *Methanosaeta* (3.52%), *Candidatus methanofastidiosum* (1.55%), and *Methanobacterium* (2.17%) became the dominant archaea in the AnMBR-AGS (Fig. 8d). In the AnMBR-AGS, acetoclastic methanogens (*Methanosaeta*, 3.52%) and hydrogenotrophic methanogens (*Candidatus methanofastidiosum* and *Methanobacterium*, 3.72%) were of similar abundance. The synthetic wastewater in this study contained high concentrations of ammonia (200 and 500 mg-N/L), and hydrogenotrophic methanogens can better adapt to a high-ammonia environment [42]. Notably, the abundance of *Geobacter* in the AnMBR-AGS was significantly higher than that in the seeding AGS (3.49% vs. 0.23%). Yan et al. [43] found that *Geobacter* could interact with *Methanosaeta* via direct interspecific electron transfer under specific conditions, such as the presence of conductive materials and ammonia-stressed anaerobic systems.

Two AnAOB and four AerAOB genera were identified in the FTBR/CANON samples, and the dominant AnAOB and AerAOB genera were *Candidatus* Kuenenia and *Nitrosomonas*, respectively (Fig. 8e). The abundances of *Nitrosomonas, Nitrospira* (NOB), and *Candidatus* Kuenenia in the CANON-biofilm (33.95%, 1.24%, and 8.88%, respectively) were higher than that in the CANON-sup (9.56%, 0.03%, and 0.98%, respectively), whereas the total abundance of denitrifiers in the CANON-biofilm (18.91%) was significantly lower than that in the CANON-sup (41.10%). The CANON reaction resulted in high concentrations of NO_3_^−^-N in the supernatant (Fig. 2c), and microorganisms in the supernatant were primarily exposed to COD from the influent. This led to an enrichment of denitrifiers in the FTBR/CANON supernatant. The denitrifiers in the CANON supernatant utilized the influent COD to reduce the NO_3_^−^-N concentration, thereby enhancing the TNRE of the combined system [44]. In contrast, continuous pump suction resulted in an infinite residence time for microorganisms on the honeycomb cotton membrane modules, resulting in the proliferation of slow-growing autotrophic bacteria. The enrichment of autotrophic bacteria also resulted in a relatively low microbial diversity in the CANON-biofilm (Table 2). Moreover, *Dechlorobacter* (20.54%) was the predominant denitrifier in the CANON-sup, whereas *Denitratisoma* (11.18%) dominated the denitrifier community in the CANON-biofilm (Fig. 8e). *Denitratisoma* is a prevalent denitrifier in autotrophic nitrogen removal systems [45,46], exhibiting intricate interactions with AnAOB. For instance, *Denitratisoma* can utilize the EPS and cell debris of AnAOB, and the partial denitrification driven by *Denitratisoma* provides NO_2_^−^-N for AnAOB [47]. Therefore, the presence of *Denitratisoma* can be used as a marker of the anammox reaction to some extent.

### 3.6 Implications for high-strength wastewater treatment

Some wastewater streams have high NH_4_^+^-N concentrations and low C/N ratio [48,49]. Conventional activated sludge processes fail to achieve efficient nitrogen removal or lose a large amount of carbon sources in the influent [50]. Moreover, according to the Monod equation [51], denitrifiers exhibit high cellular proliferation rates under high COD concentrations. In this study, integration of the AnMBR and FTBR/CANON reactors enabled simultaneous CH_4_ production and TN removal, aligning with the concept of energy-neutral wastewater treatment [52]. During the 155-day operation period, no supplementary carbon sources were added, and no excess biomass was discharged from the combined system. The AnMBR-FTBR/CANON process could recover biomass energy (CH_4_) with low excess sludge production, low land requirements, and low operation costs, implying that it is a potential energy-neutral process. Dai et al. [53] used a lab-scale CANON-MBR reactor to treat the AnMBR effluent. Owing to the use of a flat-sheet ultrafiltration membrane module, regular cleaning was an inevitable requirement for the CANON-MBR. However, in this study, the TMP of the FTBR/CANON reactor was maintained at a considerably low level (∼2 kPa) over the entire operation period. Compared with traditional membranes, the honeycomb cotton modules utilized in this study exhibited superior antifouling performance; thus, the FTBR could serve as an alternative for the CANON process. Moreover, similar to a traditional biofilm reactor, the honeycomb cotton modules used in this study can act as biocarriers, allowing the colonization of functional bacteria. In future studies, it will be imperative to optimize the reactor configuration and operational parameters of the FTBR/CANON process in accordance with the practical requirements to lay a solid foundation for broad applications.

## 4 Conclusions

A novel simultaneous nitrogen and carbon removal process was developed by integrating an external AnMBR with an FTBR. The combined system achieved satisfactory carbon and nitrogen removal as well as biogas recovery. The main conclusions of this study are summarized as follows:

(1) The combined process exhibited excellent performance in the removal of COD and TN. With influent NH_4_^+^-N concentrations of 200 and 500 mg-N/L, the average COD removal efficiencies were 98.5% and 98.3%, respectively, whereas those for TN were 64.2% and 76.4%, respectively.
(2) Over 90% of the COD was removed by the AnMBR. Nearly 50% of the influent COD was converted into gaseous CH_4_ in the AnMBR. Furthermore, approximately 59.6–74.8% of the influent TN was successfully removed by the FTBR/CANON. Anammox contributed to more than 80% of the TN elimination.
(3) *Methanosaeta* (3.52%), *Candidatus Methanofastidiosum* (1.55%), and *Methanobacterium* (2.17%) were the predominant methanogens in the AnMBR granules. In the CANON-biofilm, *Nitrosomonas* (34.0%) and *Candidatus* Kuenenia (8.88%) were the predominant AOB and AnAOB, respectively.

## Data Availability Statement

All data generated or analyzed during this study are included in this published article and its supplementary information files or are available upon request.

## CRediT authorship contribution statement

**Xueshen Wu:** Writing – original draft, Investigation, Formal analysis, Data curation. **Chao Wang:** Methodology, Data curation. **Depeng Wang:** Methodology, Formal analysis, Data curation. **Ahmed Tawfik:** Writing – review & editing. **Ronghua Xu:** Methodology, Data curation. **Zhong Yu:** Methodology, Formal analysis, Data curation. **Fangang Meng:** Writing – review & editing, Supervision, Methodology, Funding acquisition.

## Declaration of Competing Interest

The authors declare that they have no known competing financial interests or personal relationships that could have appeared to influence the work reported in this paper.

## Appendix Supplementary materials

Image, application 1
