# Supplementary material for: Achieving simultaneous removal of carbon and nitrogen by an integrated process of anaerobic membrane bioreactor and flow-through biofilm reactor
Source: Eng Microbiol. 2023 Dec 15;4(1):100136. doi: 10.1016/j.engmic.2023.100136 (PMC11610988; doi:10.1016/j.engmic.2023.100136)
Supplement: Supplementary file 1 [file mmc1.docx]

**Supporting Information**

**Achieving simultaneous removal of carbon and nitrogen by an integrated process of anaerobic membrane bioreactor and flow-through biofilm reactor**

Xueshen Wu ^a,b^, Chao Wang ^a,b^, Depeng Wang ^a,b^, Ahmed Tawfik ^c,d^, Ronghua Xu ^a,b^, Zhong Yu ^a,b^, and Fangang Meng ^a,b*^

^a^ School of Environmental Science and Engineering, Sun Yat-sen University, Guangzhou 510006, PR China

^b^ Guangdong Provincial Key Laboratory of Environmental Pollution Control and Remediation Technology, Guangzhou 510006, PR China

^c^ National Research Centre, Water Pollution Research Department, 12622, Dokki, Cairo, Egypt

^d^ College of Life Sciences, Environmental Sciences Department, Kuwait University, P.O.5969, Safat 13060, Kuwait

*Corresponding author.

Tel.: +86 (20) 39332758; fax: +86 (20) 39332742

E-mail address: mengfg@mail.sysu.edu.cn

Table S1 Chemical composition of the synthetic livestock wastewater

| Substrates | mg/L | Trace elements | mg/L |
| --- | --- | --- | --- |
| Glucose | 935（0 at phase I） | EDTA | 20 |
| Na-acetate | 1282（0 at phase I） | FeSO_4_·7H_2_O | 2.5 |
| Yeast extract | 67（0 at phase I） | ZnCl_2_ | 0.06 |
| NH_4_Cl | 764 （Phase I）  764（Phase II）  1910（Phase III） | MnCl_2_·4H_2_O | 0.06 |
| KH_2_PO_4_ | 88 | NaMoO_4_·2H_2_O | 0.19 |
| Na_2_CO_3_ | 200 | CoCl_2_·6H_2_O | 0.13 |
|  |  | NiCl_2_·4H_2_O | 0.04 |
|  |  | CuSO_4_ | 0.06 |
|  |  | CoCl_2_ | 0.44 |
|  |  | H_3_BO_3_ | 0.06 |
|  |  | MgCl_2_·6H_2_O | 0.19 |

Table S2 Some details of AnMBR

|  | value |  | value |
| --- | --- | --- | --- |
| HRT | 1 d | Membrane area | 0.021 m^2^ |
| Volume (EGSB) | 5 L | Pore size | 0.05 *μ*m |
| MLSS | 8000-10000 mg/L | Membrane flux | 10 LMH |
| Ratio of height to diameter (H/D) for EGSB | 12.5 | Membrane material | PVDF |
| Internal recirculation ratio | 800% | Biogas sparging rate | 3 L/min |
| Operating temperature (EGSB) | 30℃ | Operating temperature  (membrane tank) | 20-25℃ |
| Upflow velocity of EGSB | 37 cm/h | Volume (membrane tank) | 5 L |

**Figure S1.** DO concentration during an aeration cycle in FTBR/CANON reactor (Phase III).


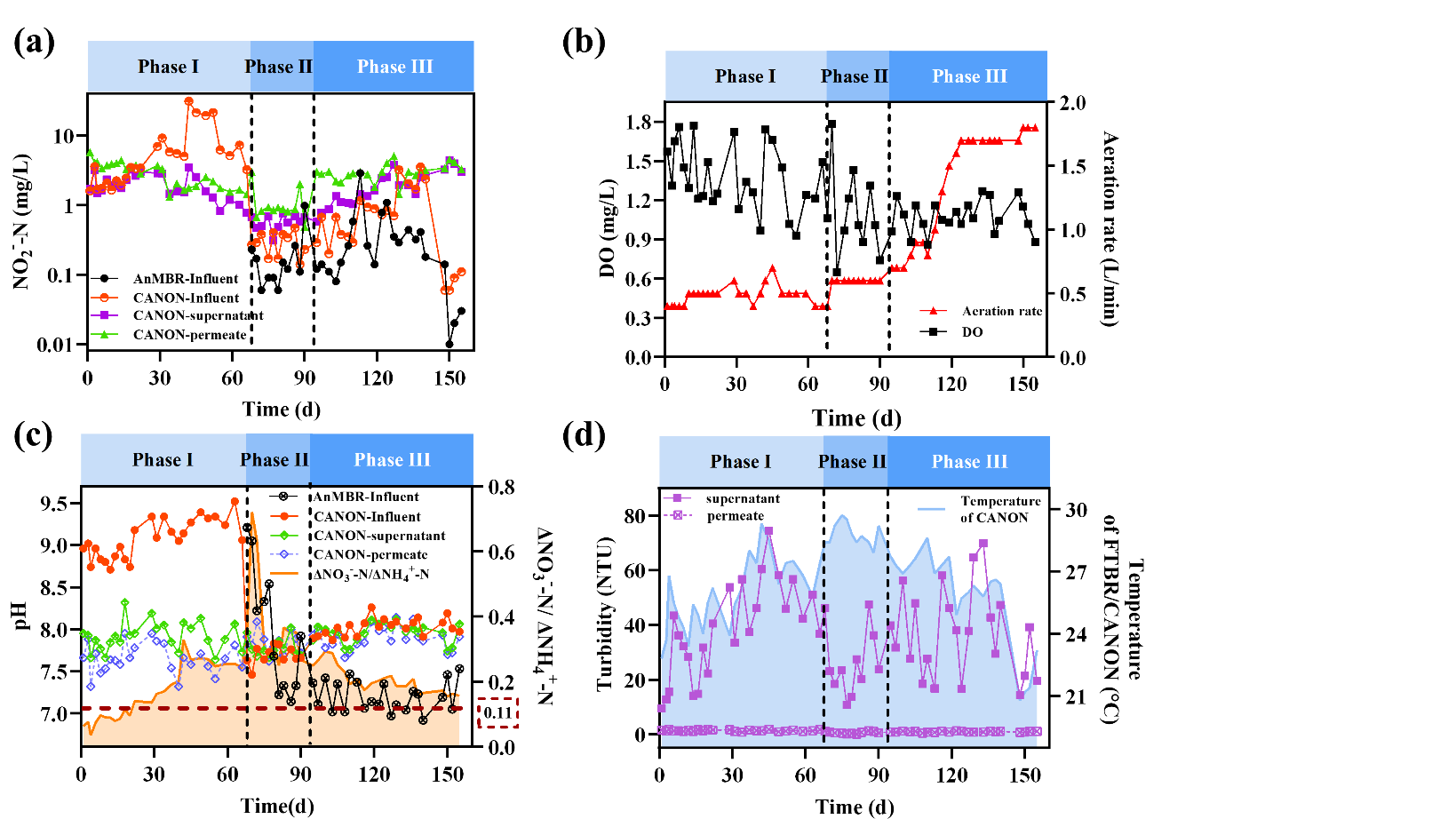


**Figure S2.** The concentration of NO_2_^‑^-N (a); DO and aerobic rate (b); pH and ∆NO_3_^-^-N/∆NH_4_^+^-N (c); turbidity and temperature of AnMBR-FTBR/CANON process.
